# The promise of dual targeting Akt/mTOR signaling in lethal prostate cancer

**DOI:** 10.18632/oncotarget.771

**Published:** 2012-12-05

**Authors:** Nicolas Floc'h, Cory Abate-Shen

**Affiliations:** Departments of Urology and Pathology and Cell Biology, Herbert Irving Comprehensive Cancer Center, Columbia University Medical Center, New York, NY; Departments of Urology and Pathology and Cell Biology, Herbert Irving Comprehensive Cancer Center, Columbia University Medical Center, New York, NY

According to the American Cancer Society approximately 242,000 new cases of prostate cancer will be reported in 2012, making it one of the most frequently diagnosed cancers in men. Moreover, with 1 in 9 men diagnosed with prostate cancer anticipated to die of the disease, prostate cancer is also the second-leading cause of cancer death in men. Although several new treatment options for men with advanced prostate cancer have recently become available, none are as yet curative.

Because of the necessity of androgen receptor signaling for all aspects of normal prostate growth, as well as for prostate tumorigenesis, androgen deprivation therapy (*i.e.,* pharmacological depletion of androgens) has long been a mainstay for treatment of men with advanced prostate cancer. However, while androgen deprivation initially leads to tumor regression, ultimately it leads to a highly aggressive form of the disease, which has been called “castration-resistant prostate cancer” (CRPC) to indicate its continued dependence on androgen receptor signaling despite the absence of androgens [1].

The standard of care for CRPC is chemotherapy, which provides a modest improvement in survival but is not curative [2, 3]. In the past few years, several new treatment options for CRPC have received FDA approval, including an immunotherapy agent, namely sipuleucel-T (PROVENGE®), and two agents that inhibit androgen receptor signaling or activity, namely Abiraterone Acetate (ZytigaTM) and enzalutamide (XTANDI®) [4-6]. Although each of these has a significant survival benefit, none are curative for CRPC. Therefore an important challenge is to identify new therapeutic approaches that are curative for CRPC.

**Figure 1:**
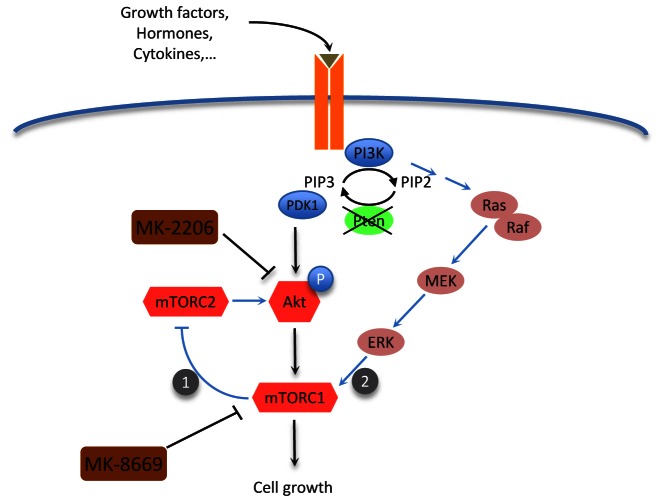
Cross talk between Akt/mTOR and MEK signaling pathways Loss of function of *PTEN* leads to activation of the mTOR pathway mainly via the PI3K/Akt pathway and secondarily via the MEK pathway. Inhibition of mTOR using MK-8669 leads to a feedback loop activation of Akt (1) Our findings suggest that inhibition of Akt using MK-2206 is not effective for inhibition of mTOR because of MEK pathway activation (2). Dual inhibition of both with MK-8669 and MK-2206 leads to effective inhibition of Akt/mTOR signaling.

As an alternative to inhibiting androgen receptor signaling, agents that target critical survival and proliferative pathways that are deregulated in prostate cancer are also being investigated. Among the molecular pathways that are critical for prostate tumorigenesis, loss of function of the *PTEN* tumor suppressor through its deletion, mutation, or deregulated expression occurs at high frequency [7], leading to aberrant activation of the Akt kinase as well as it downstream effector, the mTOR complex (Figure 1). Notably, the Akt/mTOR signaling pathway is frequently deregulated in advanced prostate cancer and has been implicated in potentiating androgen receptor signaling; however, targeting this pathway with single agents has proven not to be effective in prostate cancer, as well as is the case for many other cancers [8].

Consequently, in our recent study we asked whether combined inhibition of upstream and downstream nodes in the Akt/mTOR signaling pathway is effective for suppressing CRPC [9]. We used both *in vivo* studies of a genetically-engineered mouse (GEM) model of CRPC and *in vitro* studies using human prostate cancer cell lines to investigate the consequences of dual targeting Akt/mTOR signaling, with an inhibitor of Akt, namely, MK-2206, and one that targets mTORC1, namely ridaforolimus (MK-8669) (Figure 1). We found that dual inhibition of Akt and mTORC1 had very promising therapeutic outcomes both in the preclinical studies *in vivo* in the GEM models and in the human prostate cancer cell lines *in vitro*.

Why would dual targeting of two kinases in the same pathway yield improved results compared with targeting the pathways individually? In part, this reflects the fact that inhibition of mTORC1 alone has limited efficacy, in part due to feedback activation of PI3K/Akt signaling (Figure 1), particularly in human prostate cancer cells [9]. On the other hand, inhibition of Akt alone was inefficient for inhibition of mTOR, likely due to MAPK signaling-dependent activation of mTOR (Figure 1). Consequently, by combining two distinct inhibitors (“vertical inhibition”) we observed an effective neutralization of the Akt/mTOR pathway, which was associated with reduced cell proliferation and inhibition of CRPC tumor growth.

How might these findings be applied in the clinic? Currently, several studies have described a crosstalk between the PI3K/Akt/mTOR and AR signaling pathways. In particular, it has been suggested that castration-resistant tumors compensate for reduced AR signaling by activation of Akt/mTOR signaling, which highlights the therapeutic potential of combining inhibition of Akt/mTOR pathways with androgen receptor pathway inhibition. Indeed, Carver et al have suggested that inhibition of the PI3K pathway restores AR signaling in PTEN-deficient prostate cells, and that feedback regulation between AR and PI3K pathways may be reciprocal [10].

Thus, based on recent findings by us and others, we propose that a therapeutic approach using dual inhibitors of Akt/mTOR signaling in combination with inhibitors of androgen receptor signaling, such as Abiraterone Acetate or Enzalutamide, may effectively block androgen signaling while simultaneously inhibiting a Akt/mTOR signaling and therefore an effective treatment course for CRPC. We await the results of the relevant clinical trials.
